# A conditional glutamatergic synaptic vesicle marker for *Drosophila*

**DOI:** 10.1093/g3journal/jkab453

**Published:** 2022-01-03

**Authors:** Sarah J Certel, Evelyne Ruchti, Brian D McCabe, R Steven Stowers

**Affiliations:** 1 Division of Biological Sciences, Center for Structural and Functional Neuroscience, The University of Montana, Missoula, MT 59812, USA; 2 Brain Mind Institute, Swiss Federal Institute of Technology (EPFL), Lausanne VD 1015, Switzerland; 3 Department of Microbiology and Cell Biology, Montana State University, Bozeman, MT 59717, USA

**Keywords:** *Drosophila*, vGlut, glutamatergic, synaptic vesicle, epitope tag

## Abstract

Glutamate is a principal neurotransmitter used extensively by the nervous systems of all vertebrate and invertebrate animals. It is primarily an excitatory neurotransmitter that has been implicated in nervous system development, as well as a myriad of brain functions from the simple transmission of information between neurons to more complex aspects of nervous system function including synaptic plasticity, learning, and memory. Identification of glutamatergic neurons and their sites of glutamate release are thus essential for understanding the mechanisms of neural circuit function and how information is processed to generate behavior. Here, we describe and characterize smFLAG-vGlut, a conditional marker of glutamatergic synaptic vesicles for the *Drosophila* model system. smFLAG-vGlut is validated for functionality, conditional expression, and specificity for glutamatergic neurons and synaptic vesicles. The utility of smFLAG-vGlut is demonstrated by glutamatergic neurotransmitter phenotyping of 26 different central complex neuron types of which nine were established to be glutamatergic. This illumination of glutamate neurotransmitter usage will enhance the modeling of central complex neural circuitry and thereby our understanding of information processing by this region of the fly brain. The use of smFLAG for glutamatergic neurotransmitter phenotyping and identification of glutamate release sites can be extended to any *Drosophila* neuron(s) represented by a binary transcription system driver.

## Teaser

The development and characterization of a conditional, epitope-tagged glutamatergic synaptic vesicle for the Drosophila model system is reported. The conditional glutamatergic synaptic vesicle marker, smFLAG-vGlut, is demonstrated to be conditionally expressible, specifically expressed in glutamatergic neurons, and subcellularly localized to glutamatergic synaptic vesicles. Additionally, the utility of smFLAG-vGlut is demonstrated by glutamatergic neurotransmitter phenotyping of 26 different neuron types of the central complex of which nine were determined to be glutamatergic.

## Introduction

Glutamate is one of the three major neurotransmitters used by organisms throughout the animal kingdom. The ability to identify glutamatergic neurons and define sites of glutamate release are therefore essential to understanding neural circuit function and behavior. Neurons do not have a dedicated neurotransmitter synthesis enzyme for glutamate, as exists for the two other major fast neurotransmitters acetylcholine (cholineacetyltransferase) and GABA (glutamic acid decarboxylase), that can be used as a marker for glutamatergic neurons. The established marker for the identification of glutamatergic neurons is therefore the presence of vesicular glutamate transporters (vGluts) that localize to synaptic vesicles (SVs) and fill them with glutamate (Takamori 2006; Liguz-Lecznar and Skangiel-Kramska 2007). However, it can be challenging to rely on vGlut for glutamatergic neurotransmitter phenotyping. Synaptic release sites are often far from the cell body and intermingled with densely packed synapses from many other neurons, thereby making it difficult to trace vGlut signal present at a synapse back to individual cell bodies.

To overcome this challenge, we have developed a conditional epitope-tagged variant of vGlut via CRISPR/Cas9 genome editing to enable the identification of glutamatergic neurons and distinguish glutamate release sites in the *Drosophila* model system. Genome editing was performed at the endogenous *vGlut* locus to ensure the complete regulatory region of *vGlut* is intact and thus that the epitope-tagged vGlut protein will be expressed in all glutamatergic neurons. Furthermore, expression of epitope-tagged vGlut under its own promoter minimizes the likelihood of subcellular mislocalization due to expression above endogenous levels that typically occurs with ectopic expression from binary transcription systems (GAL4, LexA, Q).

Here, we describe the development and characterization of *B2RT-STOP-B2RT-smFLAG-vGlut*. The B2 recombinase target site (*B2RT*) flanked transcription STOP cassette provides conditionality and the spaghetti monster (sm) FLAG tag confers robust signal by virtue of its 10 copies of the FLAG epitope tag (Viswanathan et al. 2015). Immunolocalization, genetic, and electrophysiology data are presented validating smFLAG-vGlut as a conditional marker for glutamatergic SVs. *B2RT-STOP-B2RT-smFLAG-vGlut* is ideal for use in combination with binary transcription system drivers that express in small neuronal subsets, especially the already extensive and continually expanding collection of split-GAL4 drivers for single neuron types (Aso et al. 2014; Robie et al. 2017; Wolff and Rubin 2018; Dolan et al. 2019; Davis et al. 2020), among others. Last, the utility of *B2RT-STOP-B2RT-smFLAG-vGlut* for glutamatergic neurotransmitter phenotyping and identification of glutamate release sites is demonstrated using a collection of split-GAL4 drivers representing single neuron types of the central complex.

## Materials and methods

### Plasmid construction

The *pCFD4-vGlut* double guide RNA plasmid was generated as previously described (Port et al. 2014) and contains guide RNA sequences *ttgatccggaggcagggg* and *cacgtgtcgcgccccccc*. The *B2RT-STOP-B2RT-smFLAG-vGlut* donor plasmid was assembled in vector *pHSG298* (Takara Biosciences) using NEBuilder HiFi (New England Biolabs). The mRuby2_smFP FLAG coding sequence was obtained from plasmid *pCAG_mRuby2_smFP FLAG* (Addgene plasmid # 59760) as a gift of Loren Looger (Viswanathan et al. 2015). The complete annotated sequence of the donor plasmid is shown in Supplementary Fig. 1.

### Genome editing

The *pCFD4-vGlut* guide RNA plasmid was co-injected with the *B2RT-STOP-B2RT-smFLAG-vGlut* donor plasmid into embryos of strain *nos-Cas9 TH_attP2* (Ren et al. 2013) by Bestgene, Inc. The surviving adults that were injected as embryos were crossed to the balancer stock *yw*; *Sp/CyO*. Approximately 100 male *CyO* progeny were crossed individually to *yw*; *vGlut^SS1^*/*CyO* and screened for failed complementation on the expectation the desired genome edit would create a lethal *vGlut* allele. Each male exhibiting failed complementation was subsequently crossed to strain *yw*; *20XUAS-DSCP-B2*; *n-syb-GAL4* and third instar larval progeny of the appropriate genotype were assessed for anti-FLAG immunostaining. A stable stock of *B2RT-STOP-B2RT-smFLAG-vGlut* was established from males showing positive immunostaining in the larval ventral nerve cord (VNC).

### Immunostaining

Larval and adult immunostaining were performed as previously described (Certel and Thor 2004; Petersen and Stowers 2011). Primary antibodies and dilution factors: The SYN (3C11) mAb 1:25 developed by E. Buchner (Klagges et al. 1996) was obtained from the Developmental Studies Hybridoma Bank, created by the NICHD of the NIH and maintained at The University of Iowa, Department of Biology, Iowa City, IA 52242. Mouse anti-vGlut (Banerjee 2021) 1:10; Rabbit anti-Syt (Littleton et al. 1993) 1:1,000; Mouse anti-mCherry (Biorbyt orb256058) 1:200; Rabbit anti-mCherry (Abcam ab213511) 1:500, Rabbit Abfinity anti-GFP (Thermo-Fisher) 1:400, Rabbit anti-HA (Cell Signaling C29F4) 1:500, and Rat anti-FLAG (Novus NBP1-06712) 1:200. Secondary antibodies and dilution factors: Donkey anti-Rat Alexa 488 (Jackson Immunoresearch 712-546-153) 1:400, Donkey anti-Mouse Alexa 488 (Jackson Immunoresearch 715-545-151) 1:400, Goat anti-Rabbit Alexa 488 (Thermo-Fisher A32731) 1:200, Donkey anti-Mouse JF549 (Novus NBP1-75119JF549) 1:200, Goat anti-Rabbit JF549 (Novus NBP1-72732JF549) 1:200, Goat anti-Rabbit JF646 (NBP1-72732JF646). Larval muscles were stained with Phalloidin 405 (Biotium CF405M).

### Germline excision

Germline excision of the STOP cassette of *B2RT-STOP-B2RT-smFLAG-vGlut* was accomplished by crossing to *yw*; *nos-GAL4*; *20XUAS-DSCP-B2*. Progeny males of the appropriate genotype were crossed to a second chromosome balancer stock to recover potential germline excision chromosomes. Progeny males resulting from the balancer cross were individually crossed to the second chromosome balancer stock again and third instar larva were screened by immunostaining for anti-FLAG immunofluorescence.

### Electrophysiology

Wandering third instar larvae were dissected in HL3.1 (0 mM Ca^2+^) saline as previously described (Feng et al. 2004; Imlach and McCabe 2009). Two-electrode voltage-clamp recordings of larval neuro-muscular glutamatergic synaptic currents were carried out using borosilicate glass electrodes (1B120F-4, World Precision Instruments, FL, USA). Voltage follower electrodes (HS-9Ax0.1) were pulled (Sutter P-1000, CA, USA) to resistances of between 25 and 30 MΩ. Current injection electrodes (HS-9Ax1) were pulled to resistances of 15–20 MΩ and filled with 3 M KCl. Suction electrodes (GC120T-10, Harvard Apparatus, Kent, UK) were fire polished to a diameter of ∼5 µm and filled with recording saline, HL3.1 (1.0 mM Ca^2+^). mEJC recordings were conducted from muscle 6, segment A3. Muscle fibers were held at −60 mV, recordings were sampled at 20 kHz and lowpass filtered at 0.1 kHz, using pClamp 11 (Molecular Devices, Sunnyvale, CA, USA). mEJC amplitude and frequency were averaged over each 2-min recording. Muscle input resistance was calculated from the muscle voltage response to a −1 nA/500 ms current pulse. Recordings were rejected for analysis if current injection was >10 nA and/or muscle input resistance was <5 MΩ.

### Fly strains

Stocks from the Bloomington *Drosophila* Stock Certer (NIH P40OD018537) were used in this study. Previously described fly strains: *20XUAS-DSCP-B2* and *B2RT-STOP-B2RT-GFP-Rab3* (Williams et al. 2019); *vGlut^SS1^* (Sherer et al. 2020), *UAS-CD8-mCherry* (BDSC # 27392); *nos-GAL4* (Tracey et al. 2000) (BDSC # 4442); MBON-6/MB434B (Aso et al. 2014); *LH2094* and *LH1900* (Dolan et al. 2019); *3XUAS-Syt-smGFP-HA*, *SS52244*, *SS02255*, *SS52245*, *SS00078*, *SS52577*, *SS54295*, *SS27853*, *SS02195*, *SS00090*, *SS02254*, *SS52490*, *SS54549*, *SS02239*, *SS52578*, *SS00117*, *SS52266*, *SS52267*, *SS04778*, *SS02198*, *SS46517*, *SS47398*, *SS47432*, *SS47384*, *SS46525*, *SS50464*, and *SS02718* (Wolff and Rubin 2018). The complete genotypes of all experimental fly strains shown in the figures can be found in Supplementary material.

## Results

### Strategic design of a conditional epitope-tagged glutamatergic SV marker

To develop a conditional glutamatergic SV marker for *Drosophila*, the endogenous genomic locus of *vGlut* was chosen for conditional epitope-tagging via CRISPR/Cas9 genome editing. Unlike mammalian species that possess three vGlut homologs expressed in distinct and overlapping neuronal subsets (Liguz-Lecznar and Skangiel-Kramska 2007; Zhang et al. 2018), *Drosophila* contains only a single *vGlut* gene (Adams et al. 2000) that is used by all glutamatergic neurons. Thus, conditional tagging of the sole *Drosophila vGlut* gene is sufficient as a glutamatergic SV marker for all *Drosophila* glutamate-expressing neurons. In addition, tagging the *vGlut* genomic locus ensures recapitulation of the complete neuronal expression pattern of *vGlut* since the entire *vGlut* regulatory region is present to direct *vGlut* expression. Finally, expression of tagged-vGlut under its genomic regulatory region should result in endogenous levels of expression and, therefore, an SV subcellular distribution that recapitulates that of native vGlut. However, the possibility exists that the addition of the epitope tag could alter the trafficking, function, or stability of vGlut.

**Fig. 1.**
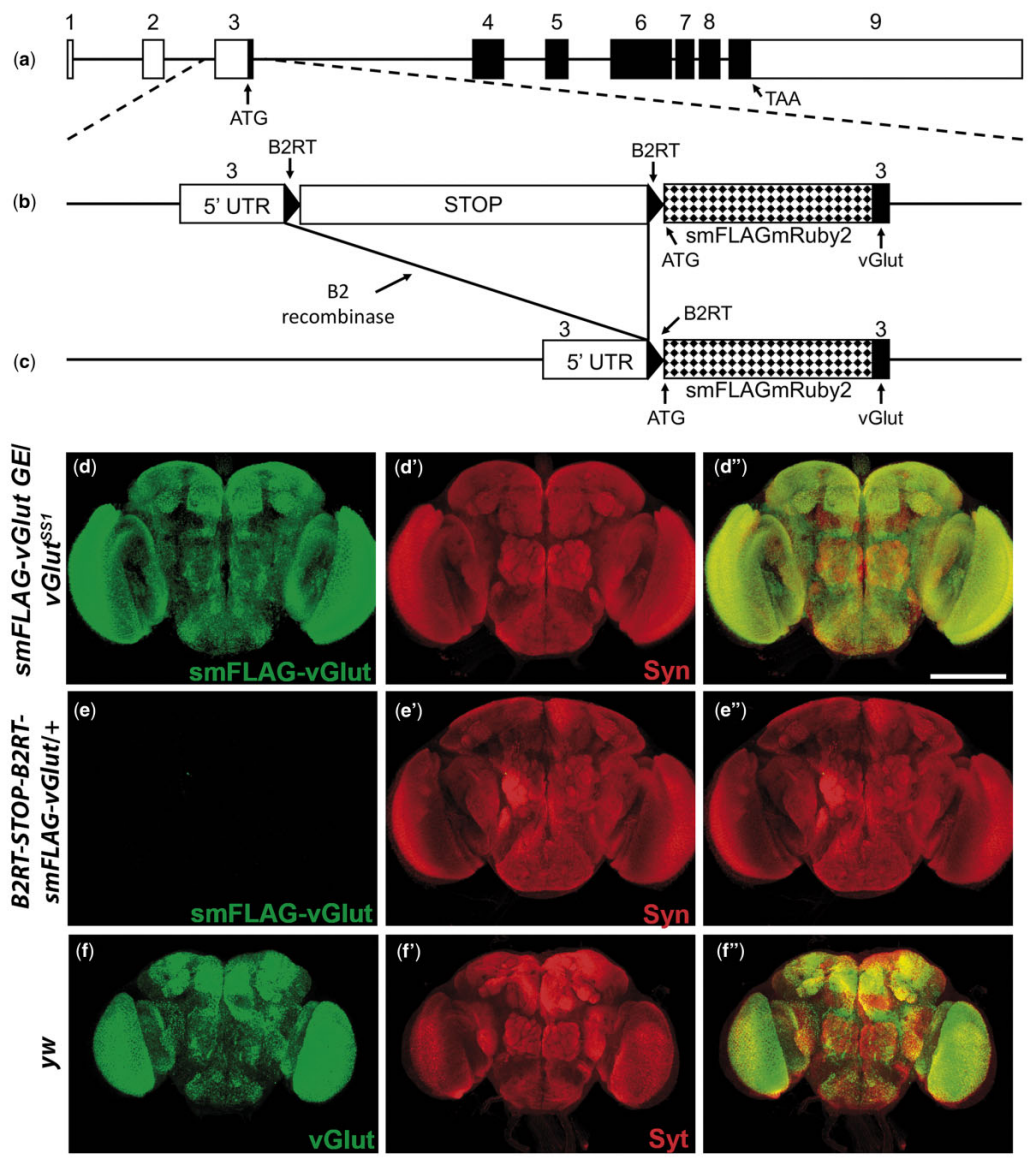
Strategic design of the conditional glutamatergic synaptic vesicle marker *B2RT-STOP-B2RT-smFLAG-vGlut* and conditional neuropil-specific expression in adult brain. a) Genomic exon structure of *Drosophila vGlut*. b) Genome editing at the endogenous *vGlut* genomic locus included insertion of a transcription STOP cassette flanked by B2 recombinase target sites (*B2RTs*) in the 5′ UTR upstream of translation start and insertion of the mRuby2_FP FLAG coding sequences in exon 3 at the ATG start codon of vGlut. Prior to excision of the STOP cassette smFLAG-vGlut is not expressed. c) After selective expression of the B2 recombinase in neurons of interest, with a binary transcription system driver and a compatible B2 recombinase responder transgene, the STOP cassette is excised and smFLAG-vGlut is expressed in glutamatergic neurons. d–e) Conditional expression in adult brain. d–d″) *smFLAG-vGlut* germline excision/*vGlut^SS1^*. d) smFLAG-vGlut; d′) Syn; d″) overlay. e–e″) *B2RT-STOP-B2RT-smFLAG-vGlut*/*+*. e) smFLAG-vGlut; e′) Syn; e″) overlay. f–f″) *yw*. f) vGlut; f′) Syt; f″) overlay. *smFLAG-vGlut* with a germline excision of the STOP cassette exhibits strong neuropil-specific immunostaining in the adult brain only after STOP cassette excision similar to endogenous vGlut. Images in (d) and (e) were collected and processed identically. Syn-Synapsin; Syt-Synaptotagmin. Scale bar: 100 µm.

The amino-terminus of vGlut was selected as the location of the epitope tag as vGlut has previously been demonstrated to tolerate amino-terminal fusion of GFP (Daniels et al. 2008). Since tagged vGlut will presumably be expressed at or near relatively low endogenous levels, and thus, detection could be challenging, mRuby2_smFP FLAG was chosen as the tag to enhance sensitivity as it contains 10 copies of the FLAG epitope (Viswanathan et al. 2015). Genome editing was targeted to the third exon of the *vGlut* gene containing the ATG start codon (Fig. 1a). A transcription STOP cassette flanked by B2 recombinase target sites (*B2RTs*) (Nern et al. 2011) was inserted in the 5′ UTR just upstream of the translation start site along with mRuby2-_smFP FLAG immediately after the endogenous vGlut ATG translation start codon (Fig. 1b). The STOP cassette prevents transcription of the mRuby2_smFP FLAG-vGlut (hereafter smFLAG-vGlut) prior to excision. However, after excision of the STOP cassette in neurons of interest via expression of the B2 recombinase, smFLAG-vGlut is expressed in neurons that are glutamatergic (Fig. 1c).

### Conditionality and SV specificity assessment

**Fig. 2.**
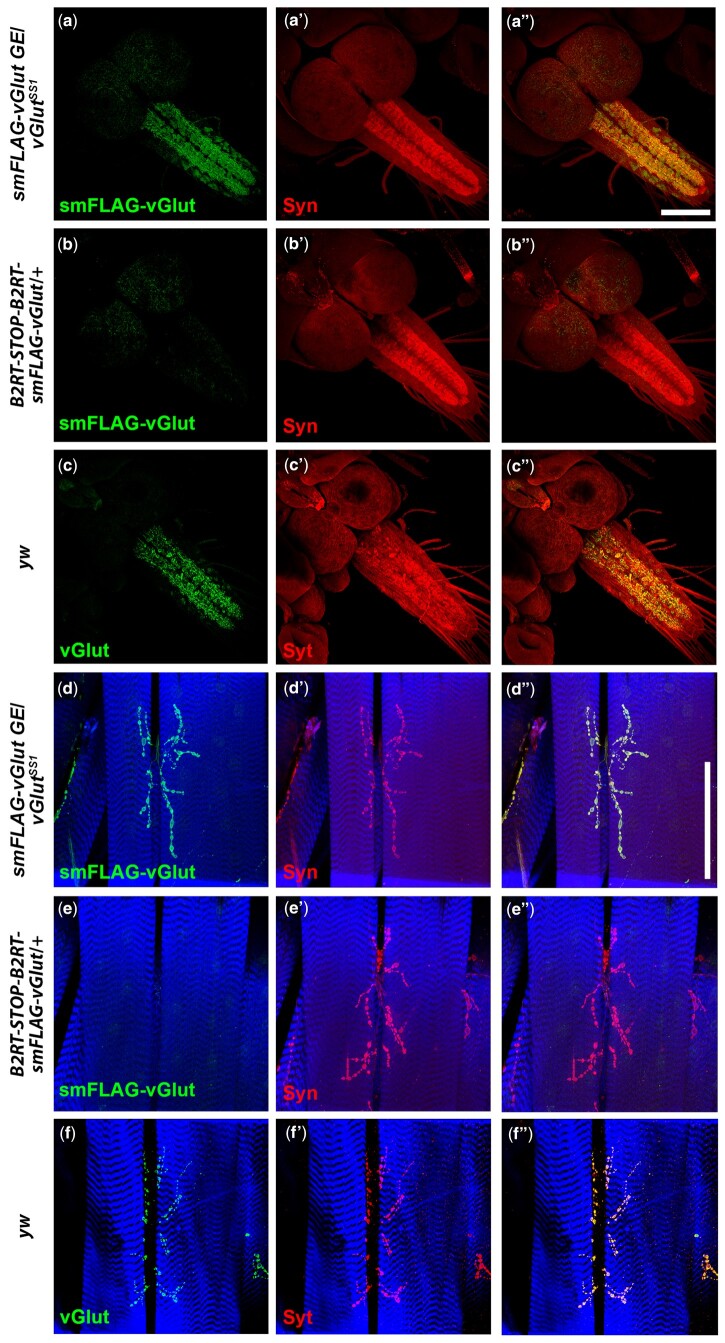
Assessment of the conditionality and synaptic vesicle specificity of *B2RT-STOP-B2RT-smFLAG-vGlut* in larval VNC and third instar larval NMJ. Third instar larval VNC. a–a″) *smFLAG-vGlut* germline excision/*vGlut^SS1^*. a) smFLAG-vGlut; a′) Syn; a″) overlay. b–b″) *B2RT-STOP-B2RT-smFLAG-vGlut*/*+*. b) smFLAG-vGlut; b′) Syn; b″) overlay. c–c″) *yw*. c) vGlut; c′) Syt; c″) overlay. Third instar larval NMJ. d–d″) *B2RT-smFLAG-vGlut* germline excision/*vGlut^SS1^*. d) smFLAG-vGlut; d′) Syn; d″) overlay. e–e″) *B2RT-STOP-B2RT-smFLAG-vGlut*/*+*. e) smFLAG-vGlut; e′) Syn; e″) overlay. f–f″) *yw*. f) vGlut; f′) Syt; f″) overlay. *smFLAG-vGlut* with a germline excision of the STOP cassette exhibits strong neuropil-specific immunostaining in the larval VNC and at presynaptic terminals of the third larval instar NMJ similar to endogenous vGlut. No detectable anti-FLAG immunostaining is observed in the larval VNC or third instar larval NMJ prior to excision of the STOP cassette. Images in (a) and (b) and images in (d) and (e) were collected and processed identically. Syn, synapsin; Syt, Synaptotagmin. Scale bars: 100 µm.

The primary intent for *B2RT-STOP-B2RT-smFLAG-vGlut* was for it to be a conditional marker of glutamatergic SVs. To evaluate conditionality, expression of smFLAG-vGlut was assessed with and without germline excision of the STOP cassette in the adult fly brain, the third instar larval VNC, and the third instar larval neuromuscular junction (NMJ). Anti-FLAG immunostaining of *smFLAG-vGlut* in adult fly brains containing a germline excision of the STOP cassette reveals robust signal broadly distributed throughout the brain (Fig. 1d). In contrast, with the STOP cassette intact no detectable anti-FLAG immunostaining was observed in *B2RT-STOP-B2RT-smFLAG-vGlut* adult fly brains (Fig. 1e). Similarly, in the third instar larval VNC (Fig. 2a) and at the third larval instar NMJ (Fig. 2d), strong signal was observed in *smFLAG-vGlut* germline excision larva, but no signal was detected with the STOP cassette present (Fig. 2, b and e, respectively). These results demonstrate the effectiveness of the transcription STOP cassette as there is no detectable constitutive expression, or “leak,” of smFLAG-vGlut in either the larval or adult nervous systems prior to excision.

To assess the specificity of smFLAG-vGlut for localization to SVs, colabeling with the SV-specific protein Synapsin (SYN) was also included in the above experiments. smFLAG-vGlut expression (Fig. 1d) was restricted to the neuropil regions of the brain as indicated by the localization of SYN (Fig. 1d′). Not unexpectedly, the distribution of SYN is somewhat broader than that of smFLAG-vGlut (Fig. 1d″) because SYN is present not just on glutamatergic SVs but on all SVs independent of neurotransmitter usage. Additional evidence consistent with the SV specificity of smFLAG-vGlut is that its expression closely resembles that of endogenous vGlut (Fig. 1f). Similarly, in the third instar larval VNC (Fig. 2a) and at the third instar larval NMJ (Fig. 2d) smFLAG-vGlut expression overlaps with SYN expression (Fig. 2, a′ and d′, respectively). These patterns of expression of smFLAG-vGlut also closely resemble that of endogenous vGlut in the larval VNC and at the third instar larval NMJ (Fig. 2, c and f, respectively).

An additional experiment was performed evaluating smFLAG-vGlut localization to glutamatergic SVs that involved simultaneous assessment of the distribution of smFLAG-vGlut and native vGlut in the same animal. Immunolabeling was performed on *smFLAG-vGlut germline excision*/*+* adult brains, larval VNCs, and larval NMJs using both anti-FLAG and anti-vGlut antibodies. Although the anti-vGlut antibody recognizes both native vGlut and smFLAG-vGlut, the anti-FLAG antibody will only recognize smFLAG-vGlut. It is thus possible there could be signal from anti-FLAG that does not overlap with signal from anti-vGlut if there is a discrepancy in expression. However, anti-FLAG and anti-vGlut immunolabeling of adult brain (Supplementary Fig. 2,a and a′, respectively), larval VNC (Supplementary Fig. 2,b and b′, respectively), and NMJ (Supplementary Fig. 2,c and c′, respectively) are nearly indistinguishable (Supplementary Fig. 2, a″, b″), and c″. Taken together, these results showing overlapping expression of smFLAG-vGlut and endogenous vGlut in the same animal, combined with results above showing similar distributions of smFLAG-vGlut and endogenous vGlut in different animals, and the overlapping expression of smFLAG-vGlut and SYN, is consistent with the specificity of smFLAG-vGlut for localization to glutamatergic SVs.

The effectiveness of the STOP cassette in suppressing expression of smFLAG-vGlut was also assessed genetically by complementation testing with the *vGlut* complete loss-of-function allele *vGlut^SS1^*. *B2RT-STOP-B2RT-smFLAG-vGlut*/*vGlut^SS1^* trans-heterozygotes were determined to be noncomplementary for *vGlut* function with an embryonic lethal phase. As it has previously been determined that *vGlut* complete loss-of-function mutants exhibit an embryonic lethal phase (Daniels et al. 2006), this result suggests *B2RT-STOP-B2RT-smFLAG-vGlut* is a complete loss-of-function, or null, allele of *vGlut*. This genetic result is consistent with the absence of detectable expression of smFLAG-vGlut when the STOP cassette is intact, as shown above.

In contrast, after germline excision of the STOP cassette, *smFLAG-vGlut*/*vGlut^SS1^* trans-heterozygotes were found to be viable (Fig. 2, a, d, and g) and fertile. The *smFLAG-vGlut* germline excision chromosome was also determined to be homozygous viable and fertile, and a *smFLAG-vGlut germline excision* homozygous stock was established that could be propagated over multiple generations. These genetic results imply smFLAG-vGlut is a functional vesicular glutamate transporter.

As an additional test of the functionality of smFLAG-vGlut, electrophysiology was performed at the third instar larval NMJ of *smFLAG-vGlut germline excision* homozygotes (*smFLAG-vGlut GE*), *smFLAG-vGlut GE* in trans to the null allele *vGlut^SS1^*, and compared to Canton-S wild-type controls. Mean spontaneous mEJC frequencies were determined to be 1.77 ± 0.31 Hz for *smFLAG-vGlut GE* homozygotes, 1.98 ± 0.32 Hz for *smFLAG-vGlut GE*/*vGlut^SS1^*, and 1.92 ± 0.35 Hz for Canton-S wildtype controls (Supplementary Fig. 3a). The mean mEJC amplitudes were 0.48 ± 0.01 nA for *smFLAG-vGlut GE* homozygotes, 0.48 ± 0.02 nA for *smFLAG-vGlut GE*/*vGlut^SS1^*, and 0.47 ± 0.02 nA for Canton-S wildtype controls (Supplementary Fig. 3b). For both measurements, no statistically significant differences were observed between any of the three genotypes for either mEJC frequency or mEJC amplitude. These electophysiology results are consistent with the genetic results above in affirming smFLAG-vGlut as a functional vGlut capable of restoring vGlut activity to mutant alleles.

### Assessment of smFLAG-vGlut for SV and neurotransmitter specificity in single neuron types

**Fig. 3.**
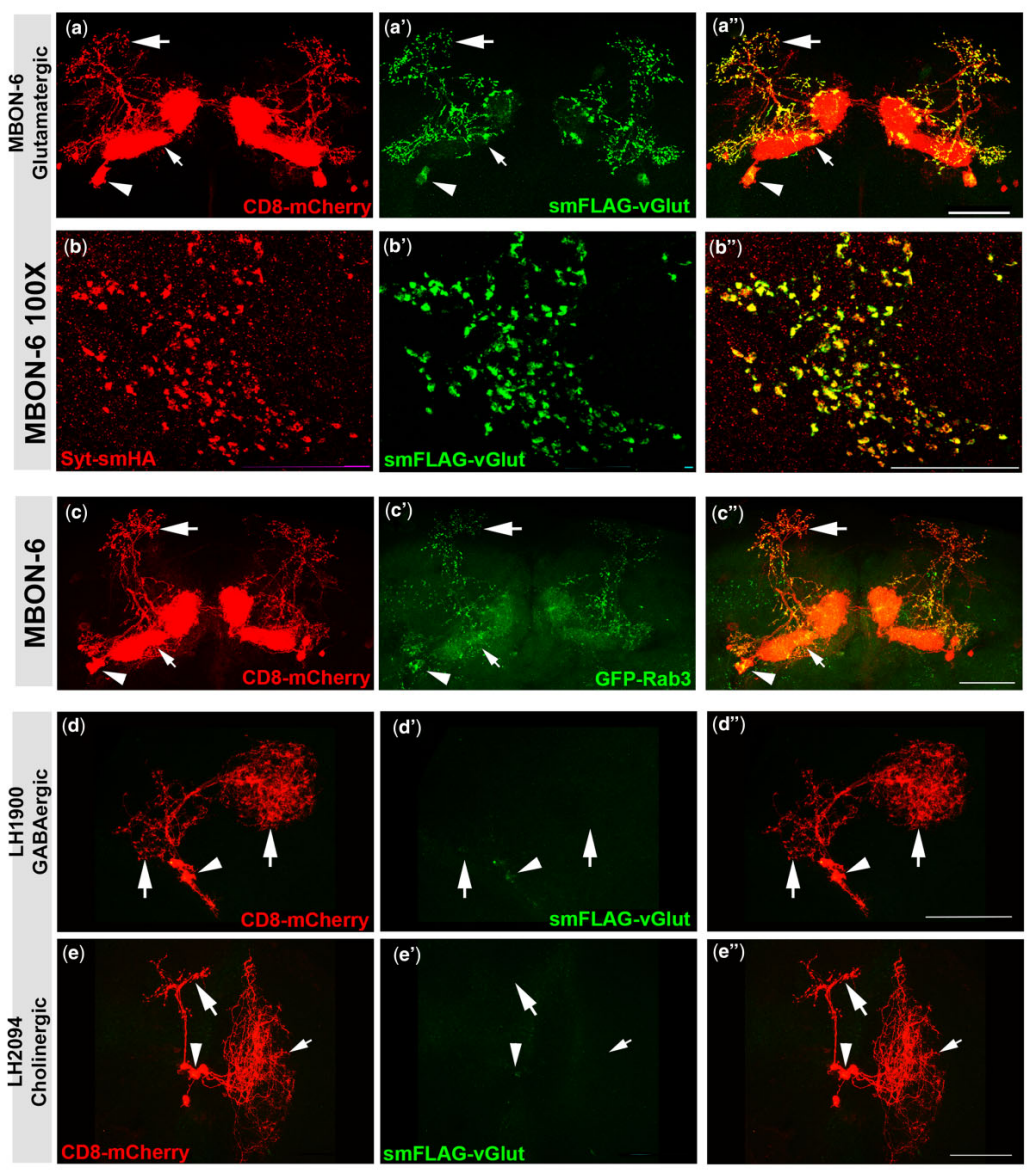
Assessment of neurotransmitter and synaptic vesicle specificity of *B2RT-STOP-B2RT-smFLAG-vGlut* in single neuron types. Neuron anatomy is visualized with the plasma membrane marker CD8-mCherry. a–a″) Glutamatergic neuron MBON-6. a) CD8-mCherry; a′) smFLAG-vGlut; a″) overlay. b–b″) High-resolution 100× images of presynaptic terminals of glutamatergic neuron MBON-6. b) Syt-smHA; b′) smFLAG-vGlut; b″) overlay. High-resolution images of MBON-6 presynaptic terminals reveal near precise overlap of smFLAG-vGlut with the synaptic vesicle marker Syt-smHA. c–c″) Glutamatergic neuron MBON-6. c) CD8-mCherry; c′) GFP-Rab3; c″) overlay. GFP-Rab3 is expressed in glutamatergic neuron MBON-6 and distributes predominantly to presynaptic terminals in a pattern highly similar to that of smFLAG-vGlut. d–d″) GABAergic neuron LH1900. d) CD8-mCherry; d′) smFLAG-vGlut; d″) overlay. e–e″) Cholinergic neuron LH2094. e) CD8-mCherry; e′) smFLAG-vGlut; e″) overlay. No expression of smFLAG-vGlut was detected in the GABAergic neuron LH1900 or the cholinergic neuron LH2094. Large arrows—presynaptic terminals; small arrows—dendrites; arrowheads—cell bodies. Scale bars: a, c, d, e)—50 µm; b)—25µm.

To determine if smFLAG-vGlut expression is specific for glutamatergic neurons and SVs, the STOP cassette of *B2RT-STOP-B2RT-smFLAG-vGlut* was excised using a 20X*UAS-DSCP-B2* recombinase transgene in combination with split-GAL4 drivers representing single neuron types of known neurotransmitter usage previously determined using independent methods. The split-GAL4 driver for mushroom body output neurons MBON-6 was selected as a positive control for glutamatergic neurons. To visualize the anatomy of each neuron of interest, the plasma membrane marker CD8-mCherry was included in this and subsequent experiments. MBON-6 is a highly polarized neuron with distinct axonal (large arrow), dendritic (small arrow), and cell body (arrowhead) regions (Fig. 3a) (Aso et al. 2014; Tison et al. 2020). smFLAG-vGlut exhibits robust signal in MBON-6 neurons and distributes predominantly to axon terminals (Fig. 3a′), similar to the distribution of the conditional SV marker GFP-Rab3 (Williams et al. 2019) (Fig. 3c′). Virtually no smFLAG-vGlut signal is observed in dendritic regions although it is detectable in the cell body. The cell body expression is presumably nascently translated smFLAG-vGlut protein awaiting transport to presynaptic terminals. High magnification (100X) images of the axon terminals of MBON-6 neurons coexpressing the SV marker Syt-smHA (Fig. 3b) and smFLAG-vGlut (Fig. 3b′) reveals nearly complete colocalization (Fig. 3b″).

Expression of smFLAG-vGlut was also assessed in the known GABAergic and cholinergic neurons LH1900 and LH2094, respectively (Dolan et al. 2019). LH1900 is a GABAergic lateral horn neuron with a distinct cell body (arrowhead) and intermingled axonal and dendritic regions (large arrows) (Tison et al. 2020) (Fig. 3d). LH2094 is a highly polarized cholinergic lateral horn neuron with distinct axonal (large arrow), dendritic (small arrow), and cell body regions (Tison et al. 2020) (Fig. 3e). Expression of smFLAG-vGlut was not detected in LH1900 (Fig. 3d′) or LH2094 (Fig. 3e′) upon excision of the STOP cassette.

Taken together, we have established smFLAG-vGLUT as a conditional marker of glutamatergic SVs by: (1) demonstrating robust smFLAG-vGlut expression in the glutamatergic neuron MBON-6 with a similar subcellular distribution as the SV marker GFP-Rab3; (2) precise spatial colocalization with the SV marker Syt-smHA; and (3) the absence of expression in GABAergic and cholinergic neurons.

### Glutamatergic neurotransmitter phenotyping of central complex neurons

With its conditionality and specificity for glutamatergic SVs thus established, the utility of *B2RT-STOP-B2RT-smFLAG-vGlut* for glutamatergic neurotransmitter phenotyping and identifying sites of glutamate release were demonstrated for 26 distinct *Drosophila* central complex neuron types of unknown neurotransmitter usage. The central complex is a key region of the insect brain involved in higher-order sensory information processing important for behavioral decision-making (Pfeiffer and Homberg 2014). In flies, a diversity of behaviors are regulated by the central complex including locomotion (Martin et al. 1999; Buchanan et al. 2015), flight (Ilius et al. 1994), courtship (Sakai and Kitamoto 2006), sleep (Donlea et al. 2014), hunger (Park et al. 2016), gravitaxis (Baker et al. 2007), and navigation (Seelig and Jayaraman 2013; Hardcastle et al. 2021). Neurons of the central complex interconnect the central complex neuropils including the protocerebral bridge, noduli, and asymmetric body, as well as surrounding neuropils (Wolff et al. 2015; Wolff and Rubin 2018; Scheffer et al. 2020). Understanding the function of this complex neuropil would be enhanced by mapping neurotransmitter usage of distinct central complex neuron types.

**Fig. 4.**
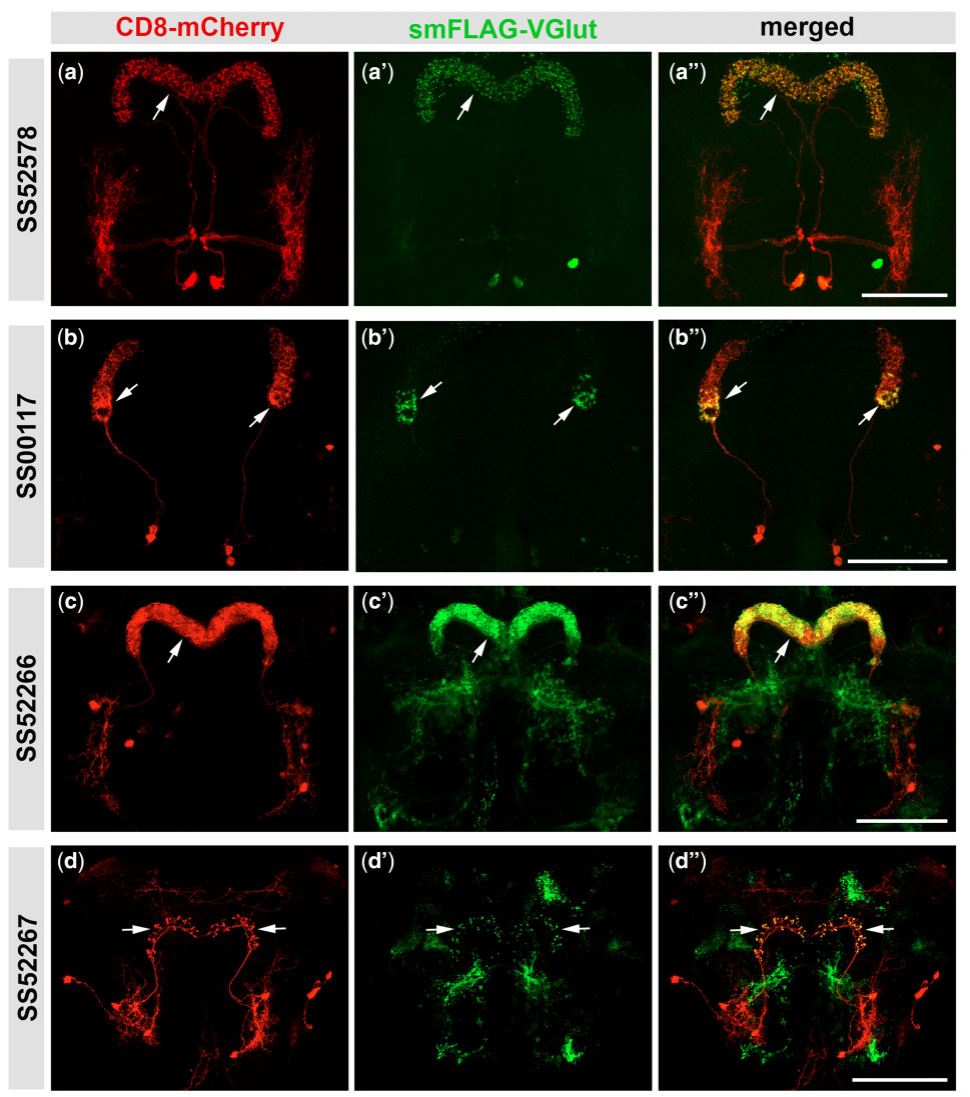
*B2RT-STOP-B2RT-smFLAG-vGlut* expression identifies glutamatergic protocerebral bridge neurons. a–a″) PB.b-LAL.s-PS.s/SS52578. b–b″) PBG_6–8_.sG_9_.b/SS00117. c–c″) PB18.s-GxΔ7 Gy.b/PB18.s-9i1i8c.b/SS52266. d–d″) PB_G1/2-9._b-SPS.s/SS52267. The plasma membrane marker CD8-mCherry (red, left column) allows visualization of the neuroanatomy of each protocerebral neuron. The presence of smFLAG-vGlut expression (arrows, middle column) indicates that each protocerebral neuron is glutamatergic and its subcellular distribution reveals the location of presynaptic terminals where SV fusion and glutamate release occurs. Scale bars: 50 µm.

**Fig. 5.**
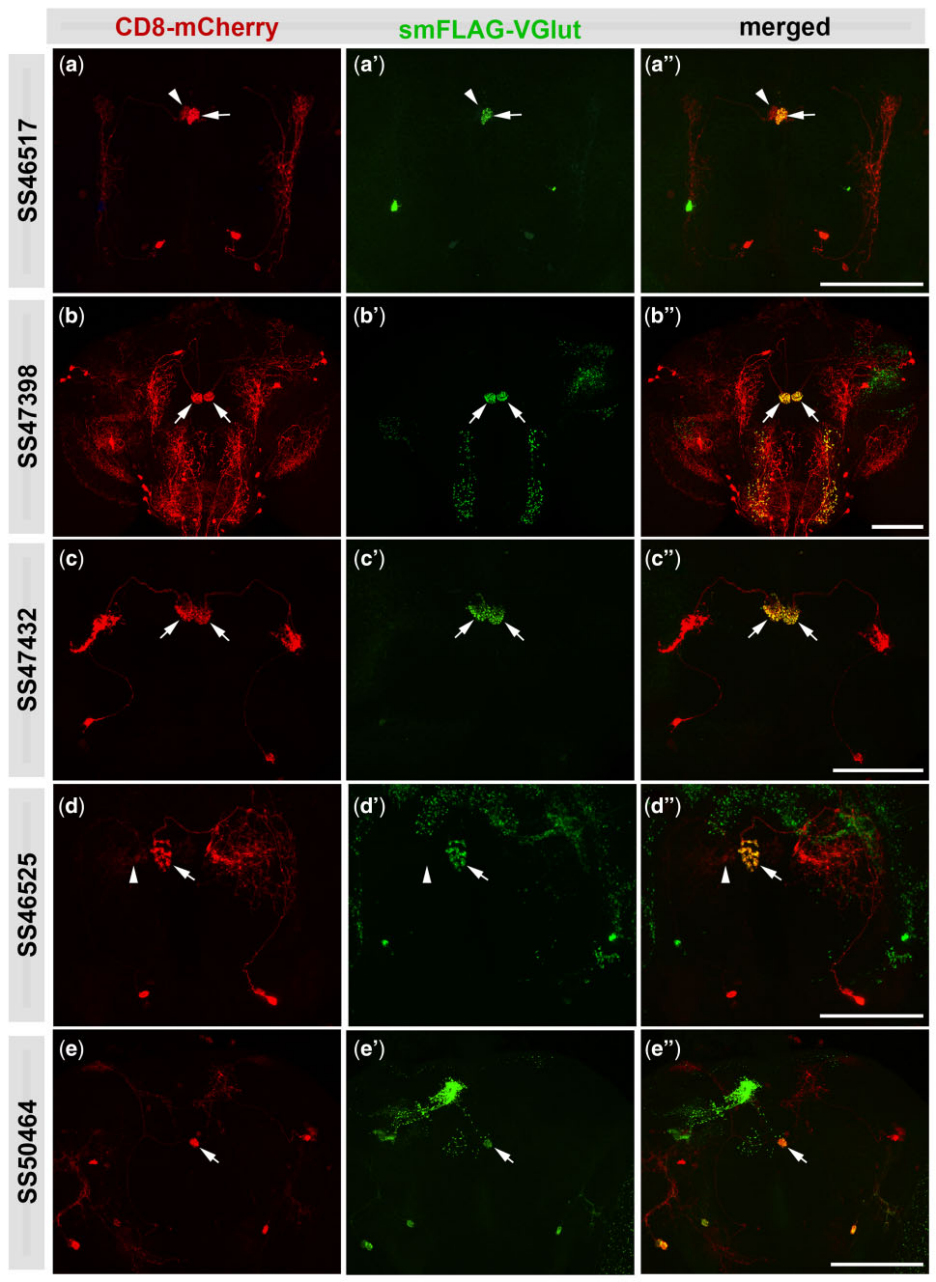
*B2RT-STOP-B2RT-smFLAG-vGlut* expression identifies glutamatergic noduli and asymmetric body neurons. a–a″) LAL.s-GAi.s-NO_1_i.b/SS46517. b–b″) LAL.s-NO_2_i.b/SS47398. c–c″) LAL.s-N0_3_Ai.b/SS47432. d–d″) LAL.s-CREc.s-NO_3_Pc.b/SS46525. e–e″) SLP-AB/SS50464. The plasma membrane marker CD8-mCherry (red, left column) allows visualization of the neuroanatomy of each neuron. The presence of smFLAG-vGlut expression (arrows, middle column) indicates that each neuron is glutamatergic and its subcellular distribution reveals the location of presynaptic terminals where SV fusion and glutamate release occur. Scale bars: 50 µm.

To determine which central complex neurons express glutamate, we took advantage of set of recently described split-GAL4 drivers representing specific neuron types of the central complex (Wolff and Rubin 2018). In total, nine of the 26 neuron types screened were glutamate positive as determined by smFLAG-vGlut expression (Figs. 4 and 5), and 17 were negative (Supplementary Fig. 4). In all images the split-Gal4-expressing neuron is visualized with the plasma membrane marker CD8-mCherry (red, left column) and axonal regions where glutamatergic SVs accumulate are indicated by smFLAG-vGlut (green, indicated with arrows, middle column). Four of the positives are protocerebral bridge neurons including PB.b-LAL.s-PS.s/SS52578 (Fig. 4, a and a″), PBG_6-8_.sG_9_.b/SS00117 (Fig. 4, b and b″), PB18.s-GxΔ7 Gy.b/PB18.s-9i1i8c.b/SS52266 (Fig. 4, c and c″), and PB_G1/2-9._b-SPS.s/SS52267 (Fig. 4, d and d″). Four additional positives are noduli neurons including LAL.s-GAi.s-NO_1_i.b/SS46517 (Fig. 5, a and a″), LAL.s-NO_2_i.b/SS47398 (Fig. 5, b and b″), LAL.s-N0_3_Ai.b/SS47432 (Fig. 5, c and c″), and LAL.s-CREc.s-NO_3_Pc.b/SS46525 (Fig. 5, d and d″) while the remaining positive neuron was the asymmetric body neuron SLP-AB/SS50464 (Fig. 5, e and e″). In the brains of some neuron types there is additional variable smFLAG-vGlut expression in neurons besides within the one(s) of interest that is discerned by the lack of overlap with the CD8-mCherry plasma membrane marker. This additional expression most likely reflects split-Gal4 driver expression during development that results in the excision of the STOP cassette prior to adulthood. While expression of the split-GAL4 driver in a given glutamatergic neuron during development may be transient and not maintained into the adult stage, any excisions in glutamatergic neurons that occurred during development will be permanent and result in smFLAG-vGlut expression.

It should also be noted that while neurons exhibiting smFLAG-vGlut expression can be considered glutamatergic with a high level of confidence, it is possible a neuron that does not express smFLAG-vGlut may still be glutamatergic. This is due to a threshold level of B2 recombinase required for excision of the STOP cassette and the level of B2 recombinase expression being dependent on the strength of the split-GAL4 driver. Thus, there is the possibility that a given neuron that scores negative for smFLAG-vGlut expression is a “false negative” because the split-GAL4 driver is not of sufficient strength to drive expression of the B2 recombinase above the threshold level required for STOP cassette excision. Examples of this are illustrated by noduli neurons LAL.s-GAi.s-NO_1_i.b/SS46517 (Fig. 5a) and LAL.s-CREc.s-NO_3_Pc.b/SS46525 (Fig. 5d) where the driver exhibits a significantly higher level of expression in the neuron on the right as compared to the one on the left. In these examples, the threshold level of expression of the B2 recombinase required for excision is only met by the neuron on the right. Consequently, smFLAG-vGlut is only observed in the presynaptic regions of the neurons on the right (arrows, Fig. 5, a′ and d′), but not the neurons on the left (arrowheads, Fig. 5, a′ and d′). Therefore, it is possible that smFLAG-vGlut expression will not be observed in some glutamatergic neurons due to weakness of the split-GAL4 driver.

## Discussion

This report describes a conditional epitope-tagged glutamatergic SV marker for *Drosophila*, *B2RT-STOP-B2RT-smFLAG-vGlut*, developed via CRISPR/Cas9 genome editing at the endogenous chromosomal locus of the single *Drosophila* vGlut. Immuno-localization data was presented demonstrating *B2RT-STOP-B2RT-smFLAG-vGlut* is conditionally expressible and specifically expressed in glutamatergic, but not cholinergic or GABAergic, neurons. Genetic analysis revealed the embryonic lethality of *B2RT-STOP-B2RT-smFLAG-vGlut*, viability of the *smFLAG-vGlut* germline excision chromosome in heterozygous combination with a *vGlut* null allele and also in homozygous condition. Electrophysiology experiments determined that spontaneous mEJP amplitude was statistically indistinguishable between smFLAG-vGliut germline excision homozygotes and wild-type controls. Together, these data strongly support our claim that *B2RT-STOP-B2RT-mFLAG-vGlut* is a conditional marker of glutamatergic SVs and a functional vesicular transporter of glutamate. smFLAG-vGlut was also shown to have a high sensitivity of detection by virtue of its robust signal even in single neurons. Finally, its utility for glutamatergic neurotransmitter phenotyping and discerning sites of glutamate release were established using split-GAL4 drivers representing 26 neuron types of the central complex, nine of which were shown to be glutamatergic.

*B2RT-STOP-B2RT-smFLAG-vGlut* provides an alternative and complementary method to single-cell transcriptomics (Henry et al. 2012; Thomas et al. 2012; Yang et al. 2016; Croset et al. 2018; Davis et al. 2020) for identifying glutamate-expressing neurons. These independent methods can potentially be used in parallel to corroborate each other’s results. Confidence in a positive glutamatergic neurotransmitter phenotype indicated by one method would be strengthened upon verification by the other method. *B2RT-STOP-B2RT-smFLAG-vGlut* does, however, have the additional advantage over single cell transcriptomics in that it provides not just whether a given neuron type is glutamatergic, but also spatial information on the location of glutamate release sites within a glutamatergic neuron. The glutamatergic neurotransmitter phenotyping utilizing *B2RT-STOP-B2RT-smFLAG-vGlut* presented herein for 26 neuron types of the central complex can be immediately extended to the current existing collection of several hundred *Drosophila* split-GAL4 drivers specific for single neuron types (Aso et al. 2014; Robie et al. 2017; Wolff and Rubin 2018; Dolan et al. 2019; Davis et al. 2020), among others, and to any additional neuron types for which split-GAL4 drivers are developed in the future.

The glutamatergic phenotype of the four protocerebral bridge neuron types, the four noduli neuron types, and the asymmetric body neuron demonstrated by expression of smFLAG-vGlut in this assessment provides useful information for modeling the neural circuitry of the central complex. Additionally, the recent report that there is no overlap in fast neurotransmitter usage in *Drosophila* (Deng et al. 2019) suggests these nine glutamatergic neurons are also not cholinergic or GABAergic. This knowledge of glutamate neurotransmitter usage in nine central complex neuron types and likely nonglutamate neurotransmitter usage in 17 other central complex neuron types, will enhance our understanding of sensory information processing and behavioral decision making in *Drosophila* central complex neurons.

While glutamate has long been known to function as an excitatory neurotransmitter in vertebrates (Curtis et al. 1959) and invertebrates (Usherwood et al. 1968), more recently it has also been shown to be an inhibitory neurotransmitter in *Drosophila* (Cully et al. 1996; Liu and Wilson 2013). The central complex neurons determined to be glutamatergic by *B2RT-STOP-B2RT-smFLAG-vGlut* thus almost certainly excite their downstream target neurons that are now comprehensively known owing to the *Drosophila* connectome (Scheffer et al. 2020), but they also have the potential to be inhibitory dependent on expression of the inhibitory glutamate receptor GluClα. To determine whether a given glutamatergic presynaptic neuron is providing inhibitory input to one of its downstream target neurons, *B2RT-STOP-B2RT-smFLAG-vGlut* could potentially be used in combination with a recently developed conditionally-expressible tagged variant of GluClα (Fendl et al. 2020). If expression of tagged GluClα is observed on the postsynapse of a downstream target neuron directly across the synaptic cleft of smFLAG-vGlut expression in a presynaptic neuron, this would provide significant evidence for inhibitory glutamatergic input.

*B2RT-STOP-B2RT-smFLAG-vGlut* is the latest addition to an existing collection of conditional tagged SV markers for *Drosophila*. This collection includes SV markers for acetylcholine (vAChT) (Pankova and Borst 2017; Tison et al. 2020), monoamines (vMAT) (Sherer et al. 2020), and the neurotransmitter-independent SV marker (Rab3) (Williams et al. 2019). *B2RT-STOP-B2RT-smFLAG-vGlut* thus fills a missing gap in *Drosophila* for a conditional glutamatergic SV marker and expands the method of neurotransmitter phenotyping by conditional expression of a tagged neurotransmitter-specific gene to include glutamate. The strategy shown herein to be successful in *Drosophila* for generating a conditional glutamatergic SV marker is potentially applicable to other species.

## Data availability

The complete annotated sequence of the *B2RT-STOP-B2RT-smFLAG-vGlut* donor construct is shown in Supplemental Information (Supplementary Fig. 1). The *B2RT-STOP-B2RT-smFLAG-vGlut* donor plasmid and *pCFD4-vGlut* guide RNA plasmid will be made available upon request. Fly strains original to this publication will be deposited at the Bloomington *Drosophila* stock center or will be made available upon request.

Supplemental material is available at *G3* online.

## Conflicts of interest statement

The authors have no conflicts of interest to declare.

## Supplementary Material

jkab453_Figure_S1

jkab453_Figure_S2

jkab453_Figure_S3

jkab453_Figure_S4

jkab453_Supplemental_Material_Legends
