# Supplementary material for: A conditional glutamatergic synaptic vesicle marker for Drosophila
Source: G3 (Bethesda). 2022 Jan 3;12(3):jkab453. doi: 10.1093/g3journal/jkab453 (PMC8895992; doi:10.1093/g3journal/jkab453)
Supplement: jkab453_Figure_S1 [file jkab453_figure_s1.docx]

**B2RT-STOP-B2RT-mruby2_smFP FLAG-vGlut**

**Tcgacggatcgttccactgagcgtcagaccccgtagaaaagatcaaaggatcttcttgagatcctttttttctgcgcgtaatctgctgcttgcaaacaaaaaaaccaccgctaccagcggtggtttgtttgccggatcaagagctaccaactctttttccgaaggtaactggcttcagcagagcgcagataccaaatactgtccttctagtgtagccgtagttaggccaccacttcaagaactctgtagcaccgcctacatacctcgctctgctaatcctgttaccagtggctgctgccagtggcgataagtcgtgtcttaccgggttggactcaagacgatagttaccggataaggcgcagcggtcgggctgaacggggggttcgtgcacacagcccagcttggagcgaacgacctacaccgaactgagatacctacagcgtgagcattgagaaagcgccacgcttcccgaagggagaaaggcggacaggtatccggtaagcggcagggtcggaacaggagagcgcacgagggagcttccagggggaaacgcctggtatctttatagtcctgtcgggtttcgccacctctgacttgagcgtcgatttttgtgatgctcgtcaggggggcggagcctatggaaaaacgccagcaacgcggcctttttacggttcctggccttttgctggccttttgctcacatgttctttcctgcgttatcccctgattctgtggataaccgtattaccgcctttgagtgagctgataccgctcgccgcagccgaacgaccgagcgcagcgagtcagtgagcgaggaagcggaagaagctcgcacattcagcagcgtttttcagcgcgttttcgatcagcgtttcaatgttggtatcaacaccaggtttaactttgaacttatcggcactgacggttactgattttgaacttttgctttgccacggaacggtctgcgttgtcgggaagatgcgtgatctgatccttcaactcagcaaaagttcgccaatacgcaaaccgcctctccccgcgcgttggccgattcattaatgcagctggcacgacaggtttcccgactggaaagcgggcagtgagcgcaacgcaattaatgtgagttagctcactcattaggcaccccaggctttacactttatgcttccggctcgtatgttgtgtggaattgtgagcggataacaatttcacaaggaaacagctatgacatgattacgaattcgagctcggtacccgcccatttgcagcgtatcgcgtagtgaataatcaagctatataatctgttatctgtgtaccttatttgtgatatcctaaccaacaacaacaaaataaattaaacgattttcgttgtctactaaaacataatttaagccaagttaatcaccaaaaatatctgtgcttaagttacgactagaggccgattatataatcttgcaccaactacgaaatggattacgtaactctggtgtggcatgtaagtaggcgttgtggccaaaggtcaaaagtacaactgttttgggggaggagcttccaatggaatcatctaaagaattttgtgaaaaacgccaaagaatgcgtacatatatactttacatttagtttcgaaattcctgatctaacaagaaataagctagtataaaaaatgcacaaacttaaaacctatgattatttccattgaatttgttttcttattataaattgtaattctaataattccaataatcgtaacattgctaactctttcgaaagagattacccaaatcaaaagaggtgtcaaatttcatgctcacaccaaatgaagcataaaacgctatcaaaaccgctcacaatgccagaaatatttacaaaatgctctgccggctcagtagttacccctcatcacaatcatttcgaaattctcgtgaatttatgaacataagcggcttacaagaaatgtgcaaaatgatgaaattgtacgagacttttgcttatcagcagcagaacacaaattgccggacccattaatagccgcatacaaatctcttttgcaggcactgtaaatgaaaatgcacaaactccacgagaatcgaaatcatatggcgaccaagtgaaaatcccccgctgagagaacgagccaacccgaaaatagagtcatcaaatttgtgctggcttcacggaaattaaccgccgccgattcgctacgcttatatacacggcaatccgccgtgaagtaggcaacaaaatatatttatgcgcccaagtgataacggcgcaaaggcaacgaagcacaacagacgctgacttgggcgaaaggacagcagcaggagcaggactcgttgatccggagggagtttcattaaggaataactaattccctaatgaaactctaacgtaagctagctagaccggtgtcgactaaagccaaatagaaaattattcagttcctggcttaagtttttaaaagtgatattatttatttggttgtaaccaaccaaaagaatgtaaataactaatacataattatgttagttttaagttagcaacaaattgattttagctatattagctacttggttaataaatagaatatatttatttaaagataattgcgtttttattgtcagggagtgagtttgcttaaaaactcgtttaggtttgtcctcccgaaattatttatttaaatgcgatggagagttggcgccgaatcgaaaactttacgcgcttaaaagcacgagttggcatccctaacgcgtaggatctttgtgaaggaaccttacttctgtggtgtgacataattggacaaactacctacagagatttaaagctctaaggtaaatataaaatttttaagtgtataatgtgttaaactactgattctaattgtttgtgtattttagattccaacctatggaactgatgaatgggagcagtggtggaatgcctttaatgaggaaaacctgttttgctcagaagaaatgccatctagtgatgatgaggctactgctgactctcaacattctactcctccaaaaaagaagagaaaggtagaagaccccaaggactttccttcagaattgctaagttttttgagtcatgctgtgtttagtaatagaactcttgcttgctttgctatttacaccacaaaggaaaaagctgcactgctatacaagaaaattatggaaaaatatttgatgtatagtgccttgactagagatcataatcagccataccacatttgtagaggttttacttgctttaaaaaacctcccacacctccccctgaacctgaaacataaaatgaatgcaattgttgttgttaacttgtttattgcagcttataatggttacaaataaagcaatagcatcacaaatttcacaaataaagcatttttttcactgcattctagttgtggtttgtccaaactcatcaatgtatcttatcatgtctggatcactagtgatctggccgggagtttcattaaggaataactaattccctaatgaaactccaggggcggagtgaggacaggcgtctgggaaaatcaggactactagctgagcagcaagttcagcatcatcagggccgttagtagccagcatcaaaccaacatggactacaaggacgacgacgacaaaggtgactacaaagatgatgacgataaaggcgactataaggacgatgacgacaagggcggaaactcactgatcaaggaaaacatgcggatgaaggtggtgatggagggctccgtgaatggtcaccagttcaagtgcaccggagagggagagggaaacccgtacatgggaactcagaccatgcgcattaaggtcatcgaaggaggtccgctgccgttcgctttcgatatcctggccacttcgttcggaggagggtcgcgcacgttcatcaagtacccgaagggaatcccggacttctttaagcagtcattcccggaaggattcacttgggaacgggtgacccggtatgaagatggaggtgtggtgactgtcatgcaagatacttcgctggaggatgggtgcctcgtgtaccacgtccaagtccgcggagtgaatttcccgtccaacggaccagtgatgcagaaaaagacgaagggttgggaacctaatactgaaatgatgtaccccgcagacggagggctgaggggctacacccacatggcgctgaaggtcgacggaggagattacaaggatgacgacgataagcaacaagattacaaagacgatgatgacaagggccagcagggcgactacaaggacgacgacgacaagcagcaggactacaaagatgacgatgataaaggaggaggacatctgtcctgttcgttcgtgaccacctacagatcaaagaaaaccgtgggaaacatcaagatgccgggcattcatgccgtcgaccaccgcctggagcggctcgaagaatcagacaatgagatgttcgtcgtgcaaagagaacatgccgtggccaagttcgcgggactgggaggcggtggaggcgattacaaagacgatgatgacaagggtgactataaagacgacgatgacaaaggggattacaaggatgatgatgataagggcggatctggcggatctggcggatctggcggatcttcattcatagccaaattgaaagccacgccattgaagggtctgacggcgtttaaggagaaggcaacgggcgtgtttggcgggtgagtcgatgtgatatgatatactctagtaacaccagacagcagggtttgggtcttaaagaatattcagatttataaaggttttttttttttaccagcaagaggatatatttaagcatagaaagcagatctatatgtatatttaaaatatatcctcattttgttggcaagtgtacagcgatgatgggtggcaatatgcccgaaatgaaatatgattgcaatcaaatggacctgcggagaacaaaagggagagagtttaatttaacgcaaggatggtcggtgctcgtggttggctgaacttgaccatgccaggataatgcagtaaatatgtaccgatattgatacgcactggcgactagccggcgtcgcgaaacttcggcacgtgtcgcgccccccccggaaagttgtaaaattattggcatgcttaacaagtcattagagggggtttatagggattctaaggacttgggacttggcacttttcactggtgacaacgaacgcagtggcatgcggacgagctcaccggtgcgtcaggaattccgggcacgttggcagcacgtgtccgggaacttcaactccggcaccttcgattgcggacgaaacgcaataaagtcgtgccactgggacatattgggcaaacactcgatgaatgtgtgtggtcgtggtatgcaatttgatgggcacacctcgtctgatttctcaaggaccttgagtgctaaattagaaattggaacaataggtggcacatcgctttcattacggaatttgttgcataccgacgaaggctgctgcagcgcttaaggcaaatgaaattgatttttacgagctgcgagccgtttccgtttccgtatccgtatccattgccaacaggacctggcgctgaagcgctcgagtggcactcggcgcgaagaaattgaaatgcctcgttgggggagtgctcaaggagttcctttatccttgctagtgaaaatgaaattagaattgccattgaagacgcggtcgtgtcgctgacaagcccccattctcccactccccctcagtgccagagcatcttcatcacagttgtcagggcaaacaaactaatacactccatacagctctacatatacatatatatctgagaatgtgccccatttacatctctaatggtcgcgttttgtggccacgttaaagtcaaaggcgaattgtacaaaagccttctgcttgtggcgccagttatccttaatctgggcggcgacaaaaagtctgcaacatttttactgacaatcaatgcggaacttgagcgccaaggatagtttggctcatcttcaaaaaatttggtgtctaaaaaatgcgacgctgatatgttctaaaatgggatcctctagagtcgacctgcaggcatgcaagcttggcactggccgtcgttttacaacgtcgtgactgggaaaaccctggcgttacccaacttaatcgccttgcagcacatccccctttcgccagctggcgtaatagcgaagaggcccgcaccgatcgcccttcccaacagttgcgcagcctgaatggcgaatgcgatttattcaacaaagccgccgtcccgtcaagtcagcgtaatgctctgccagtgttacaaccaattaaccaattctgattagaaaaactcatcgagcatcaaatgaaactgcaatttattcatatcaggattatcaataccatatttttgaaaaagccgtttctgtaatgaaggagaaaactcaccgaggcagttccataggatggcaagatcctggtatcggtctgcgattccgactcgtccaacatcaatacaacctattaatttcccctcgtcaaaaataaggttatcaagtgagaaatcaccatgagtgacgactgaatccggtgagaatggcaaaagcttatgcatttctttccagacttgttcaacaggccagccattacgctcgtcatcaaaatcactcgcatcaaccaaaccgttattcattcgtgattgcgcctgagcgagacgaaatacgcgatcgctgttaaaaggacaattacaaacaggaatcgaatgcaaccggcgcaggaacactgccagcgcatcaacaatattttcacctgaatcaggatattcttctaatacctggaatgctgttttcccggggatcgcagtggtgagtaaccatgcatcatcaggagtacggataaaatgcttgatggtcggaagaggcataaattccgtcagccagtttagtctgaccatctcatctgtaacatcattggcaacgctacctttgccatgtttcagaaacaactctggcgcatcgggcttcccatacaatcgatagattgtcgcacctgattgcccgacattatcgcgagcccatttatacccatataaatcagcatccatgttggaatttaatcgcggcttcgagcaagacgtttcccgttgaatatggctcataacaccccttgtattactgtttatgtaagcagacagttttattgttcatgatgatatatttttatcttgtgcaatgtaacatcagagattttgagacacaacgtggctttgttgaataaatcgaacttttgctgagttgaaggatcagatcacgcatcttcccgacaacgcagaccgttccgtggcaaagcaaaagttcaaaatcaccaactggtccacctacaacaaagctctcatcaaccgtggctccctcactttctggctggatgatggggcgattcaggcctggtatgagtcagcaacaccttcttcacgaggcagacctc**

**Key: guide RNA, FLAG, vGlut, B2RT, STOP, Ruby2, pHSG298.**
