# Supplementary figures and images for: A conditional glutamatergic synaptic vesicle marker for Drosophila
Source: G3 (Bethesda). 2022 Jan 3;12(3):jkab453. doi: 10.1093/g3journal/jkab453 (PMC8895992; doi:10.1093/g3journal/jkab453)

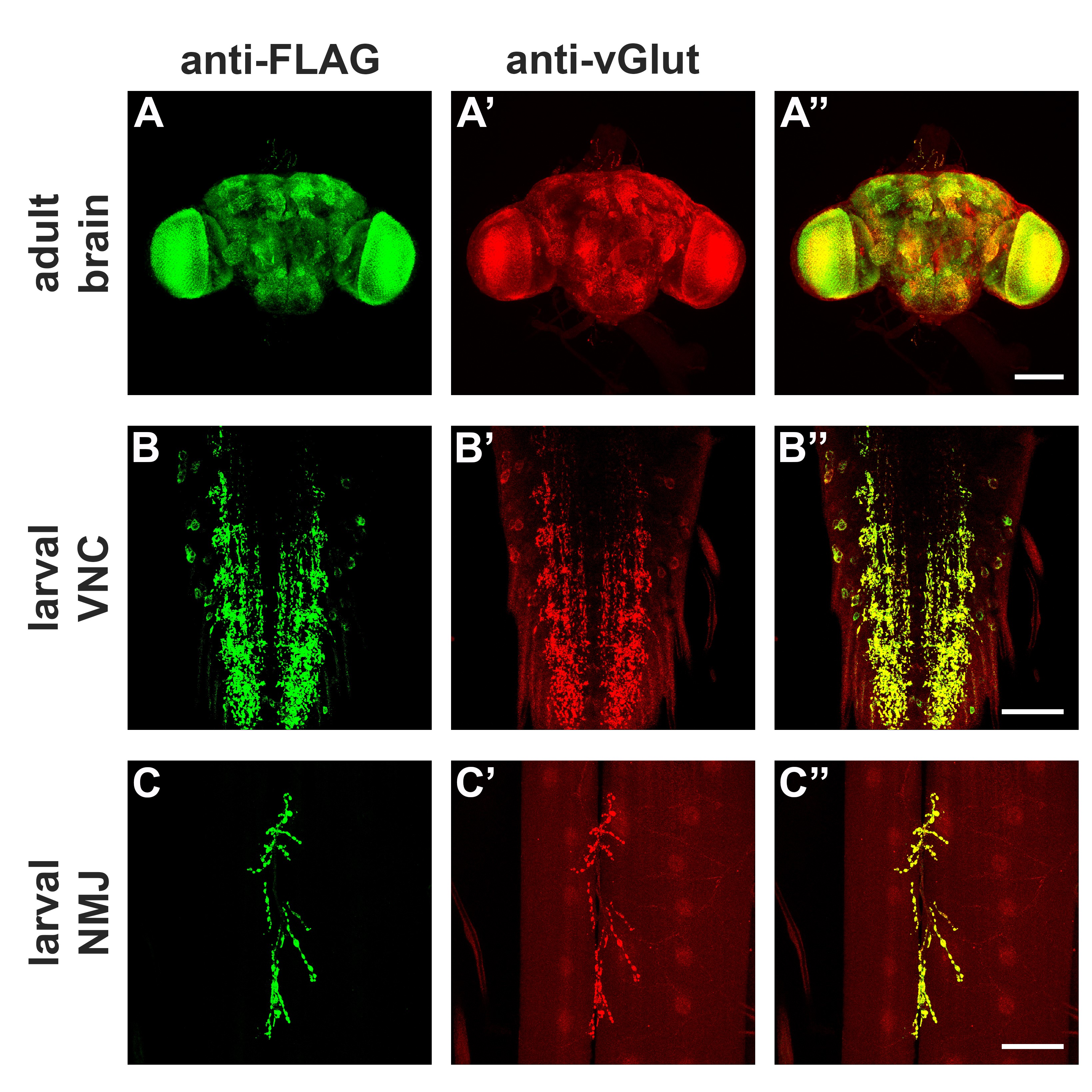

Supplement: jkab453_Figure_S2 [file jkab453_figure_s2.jpeg]

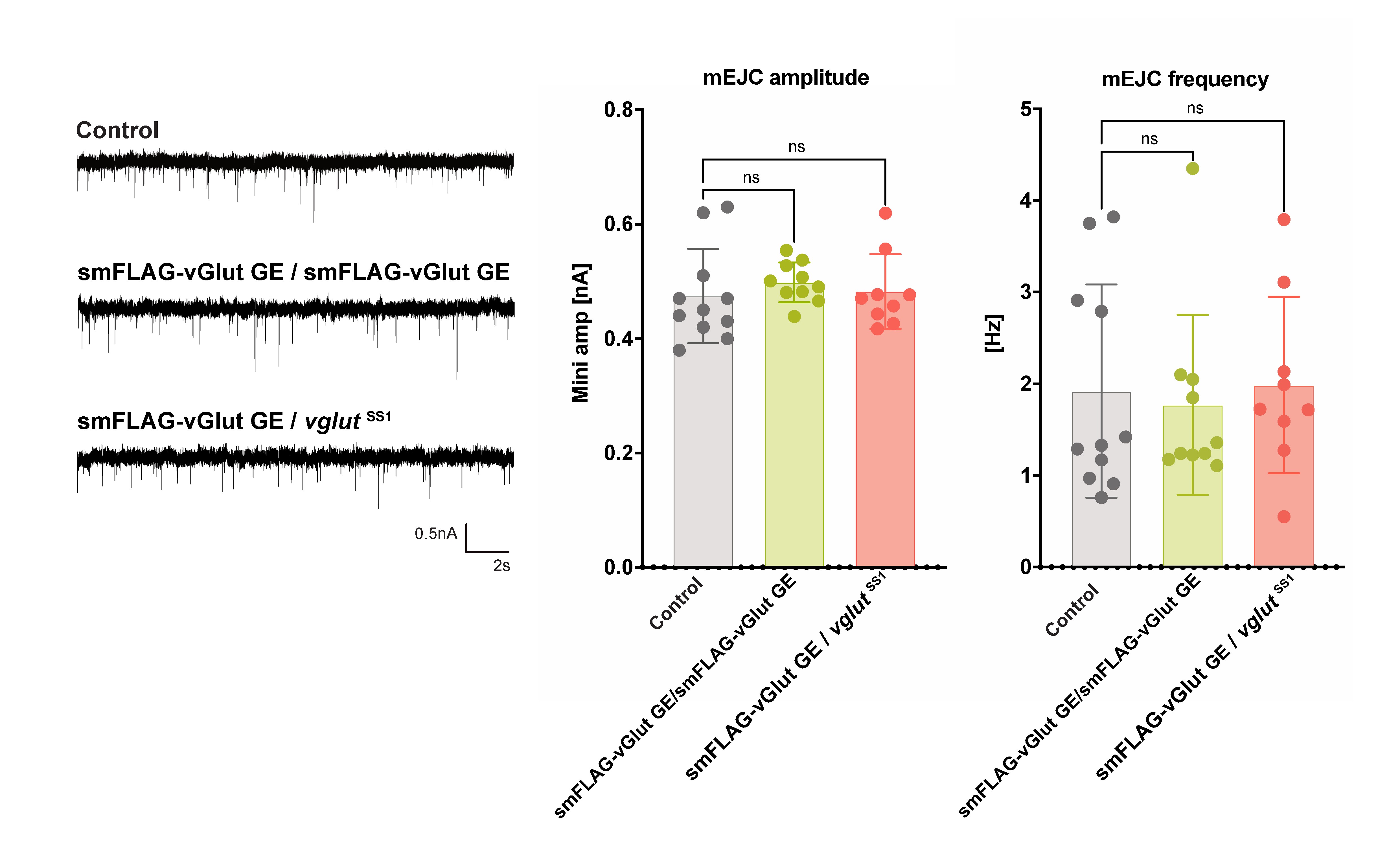

Supplement: jkab453_Figure_S3 [file jkab453_figure_s3.jpeg]

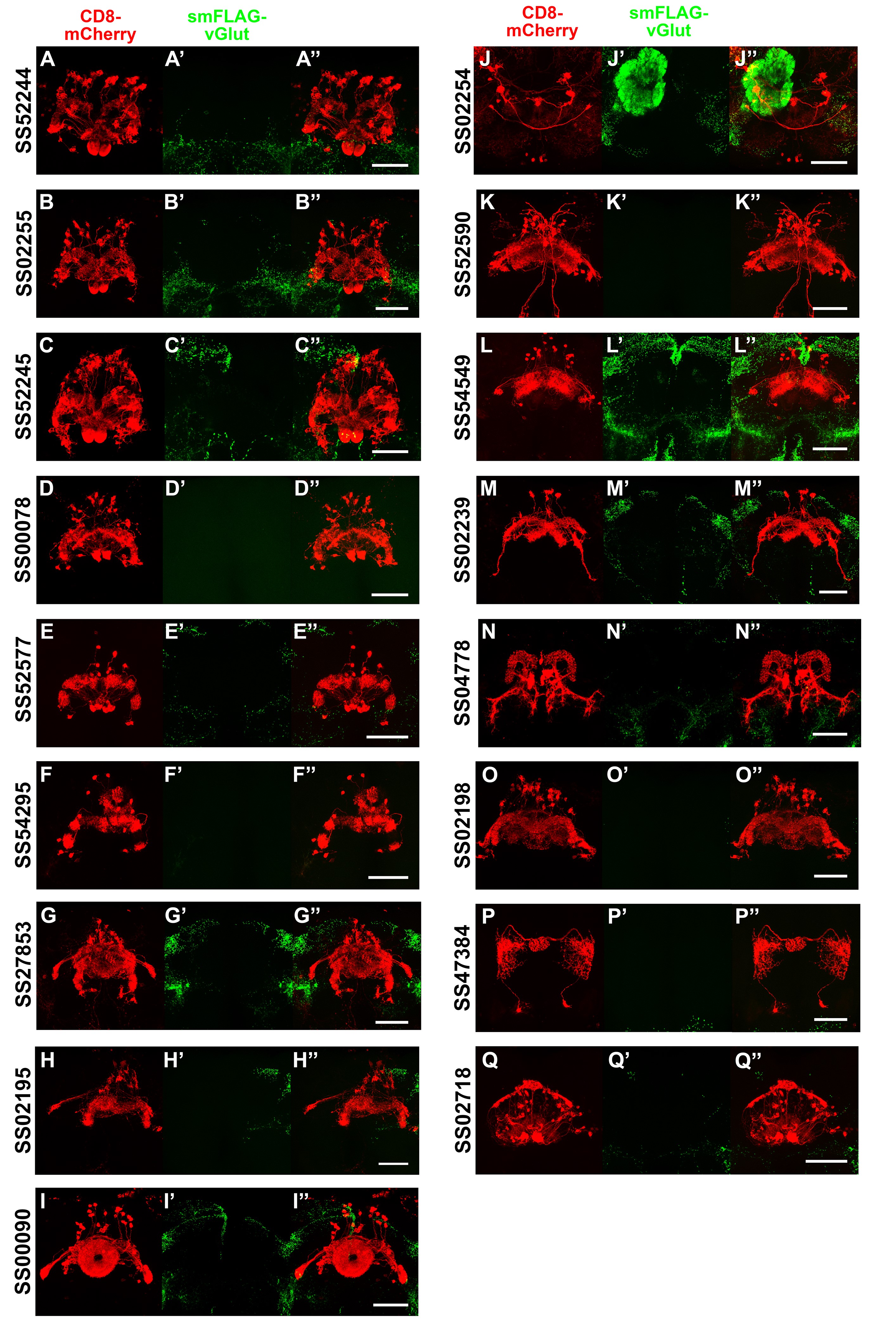

Supplement: jkab453_Figure_S4 [file jkab453_figure_s4.jpeg]
