# Supplementary material for: A conditional glutamatergic synaptic vesicle marker for Drosophila
Source: G3 (Bethesda). 2022 Jan 3;12(3):jkab453. doi: 10.1093/g3journal/jkab453 (PMC8895992; doi:10.1093/g3journal/jkab453)
Supplement: jkab453_Supplemental_Material_Legends [file jkab453_supplemental_material_legends.docx]

**Figure S1.** Annotated sequence of the *B2RT-STOP-B2RT-smFLAG-vGlut* donor plasmid.

**Figure S2.** Spatial distribution of smFLAG-vGlut and endogenous vGlut in adult brain, larval VNC, and larval NMJ of *smFLAG-vGlut germline excision*/*+* heterozygotes. A-A’’) Adult brain. A) anti-FLAG; A’) anti-vGlut; A’’) overlay. B-B’’) Third instar larval VNC. B) anti-FLAG; B’) anti-vGlut; B’’) overlay. C-C’’) Third instar larval NMJ. C) anti-FLAG; C’) anti-vGlut; C’’) overlay. The distribution of smFLAG-vGlut and endogenous vGlut is nearly indistinguishable in the adult brain, larval VNC, and larval NMJ. VNC-ventral nerve cord. NMJ-neuromuscular junction. Scale bars: A-100μm; B and C-50μm.

**Figure S3.** Electrophysiology measurements of mEJP frequency and mEJP amplitude in *smFLAG-vGlut germline excision* third instar larva. Left: Sample traces of control (*Canton-S*), *smFLAG-vGlut germline excision* homozygotes and *smFLAG-vGlut germline excision* in trans to the null allele *vGlut^SS1^*. Right: Measurement of spontaneous miniature excitatory junction current (mEJC) event amplitudes and frequency. No significant differences in these measurements between controls and either *smFLAG-vGlut* *germline excision* combination was observed.

**Figure S4.** Central complex neuron negatives for glutamatergic neurotransmitter phenotyping with *B2RT-STOP-B2RT-smFLAG-vGlut*. A-A’’) PB_G2-9_.s-FB$\mathcal{l}$1.b-NO_3_P.b/PB_G2-9_.s-FB$\mathcal{l}$1.b-NO_3_M.b/SS52244. B-B’’) PB_G2-9_.s-FB$\mathcal{l}$2.b-NO_3_A.b/SS02255. C-C’’) PB_G2-9_.s-FB$\mathcal{l}$1.b-NO_3_P.b and NO_3_M.b and NO_3_A.b/SS52245. D-D’’) PB_G2-9_.s-FB$\mathcal{l}$3.b-NO_2_D.b/SS00078. E-E’’) PB_G2-9_.s-FB$\mathcal{l}$3.b-NO_2_V.b/SS52577. F-F’’) PB_G2-9_.s-EBt.b-NO_1_.b/SS54295. G-G’’) PB_G1-9_.s-EBt.b-D/V GA.b/SS27853. H-H’’) PB_G1-9_.s-EBc.b-D/V GA.b/SS02195. I-I’’) PB_G1-8_.s-EBw.s-D/V GA.b/SS00090. J-J’’) PB_G9_.b-EB.P.s-GA-t.b/SS02254. K-K’’) PB.s-FB$\mathcal{l}$6.b-l3.s-V GA-s.b/SS52590. L-L’’) PB_G1-8_.s-FB$\mathcal{l}$3,4,5.s.b-ROB.b/SS54549. M-M’’) PB_G17_.s-FB$\mathcal{l}$2-LAL.b-cre.b/SS02239. N-N’’) PB_G2-9_.b-IB.s.SPS.s/SS04778. O-O’’) PB_G1-9_.s-EBt.b-D/V GA.b and PB_G1-9_.s-EBc.b-D/V GA.b/SS02198. P-P’’) LAL-CREi.s-NO_3_P/Mi.b/SS47384. Q-Q’’) AB-FB$\mathcal{l}$8/SS02718. The plasma membrane marker CD8-mCherry (red, left column) allows visualization of the neuroanatomy of each central complex neuron. The absence of smFLAG-vGlut expression (arrows, middle column) suggests a non-glutamatergic neurotransmitter phenotype for each neuron. Scale bars: 50μm.

**File S1.** List of genotypes associated with each figure.
